# Mapping Collaborations and Partnerships in SDG Research

**DOI:** 10.3389/frma.2020.612442

**Published:** 2021-02-09

**Authors:** Jane Payumo, Guangming He, Anusha Chintamani Manjunatha, Devin Higgins, Scout Calvert

**Affiliations:** ^1^ MSU AgBioResearch, College of Agriculture and Natural Resources, Michigan State University, East Lansing, MI, United States; ^2^ MSU Innovation Center, Michigan State University, East Lansing, MI, United States; ^3^ MSU Libraries, Michigan State University, East Lansing, MI, United States

**Keywords:** sustainable development goals, millennium development goals, collaboration, bibliometric analysis, research evaluation, CADRE, co-authorship networks

## Abstract

Collaboration has become an essential paradigm in sustainable development research and in strategies for meeting the United Nations Sustainable Development Goals (SDGs). This study uses bibliometric methods and network analysis to examine research output and collaboration supporting the SDGs and explores means to detect and analyze research collaboration beyond the traditional definition of multiple, one-time co-authorship. We employed two additional lenses of collaboration: repeat collaboration and collaboration time point to quantify and visualize co-authorship data sourced from Microsoft Academic Graph. Our results show an increased collaboration rate over time at the author and institutional levels; however they also indicate that the majority of collaborations in SDG-related research only happened once. We also found out that on average, repeat collaboration happens more frequently, but after a longer duration, at the institutional level than at the author level. For this reason, we further analyzed institutions and identified core institutions that could help influence more consistent collaboration and sustain or grow the SDG-related research network. Our results have implications for understanding sustainable partnerships in research related to SDGs and other global challenges.

## Introduction

Scientific progress in today’s research environment is increasingly driven by collaboration, a factor well-discussed in the literature (Adams, 2012; Bozeman and Boardman, 2014; Wagner, 2018). Collaboration can be defined as the “social process whereby human beings pool their human capital for the objective of producing knowledge” (Bozeman and Boardman, 2014). Collaboration is a prominent component in the calls to scientific action proposed in the 2019 Global Sustainable Development Report and will contribute to “better-aligning progress and innovation trajectories” for the United Nations 2030 Agenda for Sustainable Development (Global Sustainable Development Report, 2019). SDGs focus on a range of interrelated goals, comprising 169 targets and 232 indicators, from the eradication of poverty and inequality to taking action on climate change. The COVID-19 pandemic, which is expected to impact SDG 8 (economic growth), and SDG 3 (good health and well-being) (see e.g., United Nations, 2020), has demonstrated the need for research collaboration to accelerate new knowledge creation on new therapies and vaccines, and to share data and research results worldwide.

Since “co-authorship is one of the most tangible and well-documented forms of collaboration” in research (Glänzel and Schubert, 2005), bibliometric approaches can be used to characterize and quantify research collaboration. Studies have demonstrated a trend toward co-authorship across disciplines and research areas, domestically and internationally (e.g., Adams, 2012; Benckendorff, 2010; Bozeman and Boardman, 2014; Lee and Bozeman, 2005; Mo, 2016; Newman, 2001; Sullivan and Skelcher, 2002). The Elsevier-SciDev.net report Sustainability Science in a Global Landscape (Sustainability Science, 2015) and the 2019 Institute for Scientific Information (ISSI) report Navigating the Structure of Research on Sustainable Development Goals (Nakamura et al., 2019) used bibliometric approaches to describe trends in research outputs and collaboration related to SDGs. These reports considered research collaboration at the national and regional levels but not at the institution and author levels, which this study employs to describe the collaborative relationships involved in SDG-related research.

In order to conceptually map collaborations and partnerships in SDG research, we use bibliometric and network analysis to investigate the following questions:

• Is research activity on SDGs increasing? Is collaboration a feature of SDG research, and if so, what can we learn about changes to collaborative research networks over time?
• Do partnerships result in long-term collaborative relationships, at either author or institutional levels?
• What time intervals between publications characterize SDG research partnerships? Are partnerships sustained and do SDG research networks expand over time?
• Are SDG research networks characterized by domestic or international collaboration? Are there other features of research networks we may be able to detect?
• What role do institutions play in SDG collaborations, and can we identify key institutions that may be influential in growing and sustaining SDG research networks?

Through bibliometric and literature-based analysis, this study characterizes co-authorship of SDG-related research through measures of frequency and temporality of research collaborations, to contribute to the discussion of collaboration dynamics and sustainable, effective collaboration. This study also explores the application of network analysis and visualization tools to describe the collaborative structure of SDG-related research over time, diagnose the persistence of collaboration, and identify institutional actors that can influence the productivity of collaboration in this area. As inaugural fellows of the CADRE (Collaborative Archive & Data Research Environment) project at Indiana University (Mabry, et al., 2020), we used Microsoft Academic Graph's (MAG) bibliometric data (Sinha et al., 2015) for this study. CADRE is a multi-university project to develop a platform to curate large open and licensed datasets to allow users to construct robust queries and to save and share code, analysis steps, and data to improve the reproducibility of research outputs. MAG is one of several large datasets available on the CADRE platform.

We hypothesized that increasing attention to environmental and development concerns and institutional efforts to address sustainable development, concomitant with the evolution and articulation of the UN’s Sustainable Development Goals, would result in increased research outputs, with the potential for new patterns of collaboration across institutions and national borders. We sought to characterize those patterns of collaboration to gain insight into research partnerships with the potential to impact SDG research output.

The next two sections: “Sustainability and the Research Landscape” and “Collaboration in Context,” present a review of the concepts, rationales, and discourse on research and collaboration on SDGs that frame the approach of this study. “Materials and Methods” describes the methodology for data retrieval and analysis used to systematically profile SDG collaboration at the author and institutional levels. “Results” and “Discussion and next steps” sections follow.

### Sustainability and the Research Landscape

In this study, we characterize collaborations that conduct research on the UN's Sustainable Development Goals, or SDGs. The concept of “sustainable development” arose in the second half of the 20th century in response to debates in development discourse, encompassing both “an environmental critique and the ‘basic’ human needs” critique” of development (Macekura, 2015). As the environmental consequences of development intensified in the post-war era, tensions mounted between highly developed economies and those of the Global South over issues of the environment, economic development, and sovereignty. By the 1980s, “sustainable development” had become a common way of framing the viewpoint that environmental protection and economic development using natural resources could and should work in balance. The World Commission on Environment and Development defined sustainable development as “development that meets the needs of the present without compromising the ability of future generations to meet their own needs”) (World Commission on Environment and Development 1987). By this time, the phrase “sustainable development” had moved into the mainstream, and taken on ambiguity with overuse by NGOs, development planners, academics, and activists (Lélé, 1991).

Definitions of sustainable development abound (Lélé, 1991; Mebratu, 1998), and the concept has become relevant in a variety of domains, not just social, economic, and environmental. Despite being embraced by the mainstream, the concept of sustainable development remains powerful, and “endures as an overarching utopia, anchored in analysis of political economy and prescriptions for social change” (Baviskar, 2003). Sustainable development aims to address a multitude of phenomena from climate change, political instability, scarcity of resources, cultural conflict, economic integration, and technological innovations (Mebratu, 1998). “Sustainable development” is, thus, a flexible enough concept to foster discourse and research across many disciplines.

As successors to the Millennium Development Goals (MDGs), and the result of decades of debate and consensus-building, the Sustainable Development Goals were adopted by the United Nations in 2015. They consist of a series of 17 goals, with associated targets and indicators, to achieve by 2030, as a blueprint to achieve a “better and more sustainable future for all” (United Nations, 2020). This blueprint anticipates partnerships and cooperation among key development actors from civil society, to higher education institutions, to business, government, NGOs, and foundations.

While progress towards SDGs involves every sector of society, knowledge generation and innovation play a key role in defining problems, identifying global priorities, and proposing solutions. To address SDGs at the institutional level, metrics have been mapped to SDG targets and indicators to encourage universities and research institutions to evaluate sustainability in their core operations and thus identify routes to act on SDGs. The Aurora Universities Network is developing a dashboard for member universities to map research outputs to the SDGs (Aurora Universities Network, 2020). Times Higher Education World University rankings now include Impact Rankings, measuring almost 860 universities in 89 countries against the Sustainable Development Goals (THE Impact Ratings, 2020). Using publication data supplied by Elsevier, the rankings reported that 13% of the publications produced by these universities relate to the 17 SDGs (THE Impact Ratings, 2020).

Anticipating the inauguration and rise of “sustainable development goals” in research, Vatananan-Thesenvitz et al. (2019) used PRISMA guidelines to identify literature in Scopus that dealt with innovation in sustainable development from the time period of 1985–2018. Research conducted by an Elsevier team (Sustainability Science, 2015) investigated a broader set of measures of “sustainability science” output to inform research on sustainable development goals. Hassan et al. (2014) likewise conducted a bibliometric evaluation of sustainable development research without directly linking it to research on SDGs or MDGs.

Since 2015, there has been growth in bibliometric studies explicitly about SDG research. One study (Sweileh, 2020), using Scopus data and key terms developed by Aurora Universities Network, revealed that SDG17 (Partnerships for the Goals) was the most researched item over the past years (2015–2019). Other SDGs that dominated the overall retrieved literature included: SDGs of poverty (SDG 1), health (SDG 3), responsible consumption and production (SDG 12), climate action (SDG 12), sustainable cities and communities (SDG 11), and life on land (SDG 15). Meschede (2020), like Sweileh (2020), found that SDG 3 (“good health and well-being”) is prominent in SDG research, in an analysis of Scopus and Web of Science data that also identified major publishers and focal SDGs of the research by country of origin. Bautista-Puig and Mauleón (2019) analyzed documents about MDGs and SDGs to estimate research growth on these topics and create an ontology of key terms appearing in the literature. Nakamura et al. (2019) identified matches for “sustainable development goal(s),” expanded this core dataset with publications that cited at least one of these documents, then used bibliographic coupling to cluster the papers by themes.

### Collaboration in Context

Collaboration is an essential part of scientific research and a key element in scientific and technical human capital development (Lee and Bozeman, 2005). For some areas of research, collaboration can result in better results, achieved more quickly than when the same analysis is performed by a single researcher. Some scientific work could not be performed by an individual researcher acting alone, given that scientific practice is progressively becoming more interdisciplinary, equipment-dependent, and project-based. Robustness and reliability may be enhanced with a team of researchers evaluating the accuracy, quality, and meaning of research outcomes, with different perspectives and views, which in turn benefit institutions by developing the means to carry out their research goals. Bringing institutions and stakeholders with different agendas together may result in innovations and breakthroughs contributing to intertwined SDGs, providing more comprehensive solutions (Stibbe et al., 2018).

Different patterns of collaboration shape scientific productivity and researchers may be motivated by strategic, organizational, and operational considerations (Traoré and Landry, 1997). Strategic collaborations are motivated by shared interests and common goals (Sullivan and Skelcher, 2002). Collaboration prompted by access to expertize, equipment, technology, and funding (Bozeman and Corley, 2004; Royal Society, 2011) involves organizational and operational collaboration and generally results in research output in the form of publications (Lee and Bozeman, 2005), patents (Santamaria and Surroca, 2011) and future innovation performance (Mo, 2016; Stek and van Geenhuizen, 2016). Collaboration, particularly across institutions and national borders, exacts costs and provides benefits that institutions and researchers must weigh, including flows of funding, investments, human capital, and impact (Jeong et al., 2014).

The most typical expression of collaboration is co-authorship, when two authors co-publish a study. Some of the benefits that co-authorship brings to researchers include more credit for co-authored papers, higher impact as measured by citations, and benefits to the research itself due to the nature of teamwork (Katz and Martin, 1998). Co-authorship, especially international co-authorship, has then become a progressively dynamic area within scientometrics (Abbasi et al., 2012). In the past few decades, bibliometric studies have reported a doubling of the number of co-authors in scientific articles (Newman, 2001; Yan et al., 2010; Adams, 2012). An increasing trend toward co-authorship of publications, hence, research teamwork was observed in the field of sustainable development. Bemke-Świtilnik et al. (2020) examined collaboration trends in sustainable mining, and their results show increases in both the number of co-authored publications and in the average number of co-authors per article. At the country level, Sweileh (2020) found increased co-authored publications across all SDGs, though this varied by geographic region.

Other research has explored the characteristics of collaboration that endure. Highly persistent collaborations do not necessarily result in more impact, but moderately persistent research partnerships may show an increase in yearly average number of citations. However, the impact of persistent collaboration is also influenced by transdisciplinarity, team size, and differences in scientific age (Bu et al., 2017). Stability is another descriptor for collaborations that remain consistent from year to year, and this too may be influenced by the same factors or circumstances as persistent collaborations (Bu et al., 2018). The size and stability of research partnership cores can also be measured (Cugmas et al., 2015). Not only does the interdisciplinarity of the team interact with persistence and stability to influence impact (Bu et al., 2017; Bu et al., 2018), but the “freshness” of a team, defined as the portion of a research team who had not previously collaborated together, can result in other impacts, like disruption and influence across disciplinary boundaries (Zeng et al., 2020).

The varied motivations and experiences of collaboration suggest the need for further inquiry into patterns of co-authorship in sustainable development goal research. Traditional measures of collaboration through co-authorship allow important, but limited means for characterizing research partnerships in an urgent, globally relevant set of topics. We contribute to deepening understanding of SDG-related research partnerships by adding network analysis to the set of tools for investigating research productivity.

## Materials and Methods

This study examined the co-authorship of Sustainable Development Goal-related publications over a 20-year span (1999–2018) with data sourced from the scholarly entity graph database Microsoft Academic Graph (Sinha et al., 2015). We applied a suite of traditional bibliometric and descriptive statistics to our dataset and complemented this with network analysis and visualization. We first examined co-authorship patterns, then applied this analysis to the institutional affiliations of co-authors to examine cross-institutional collaboration.

### Bibliographic Database and Limitations

MAG is produced in part through data mining techniques developed by Microsoft Research for search engine development, and offers better coverage against other databases as confirmed in other bibliometric studies (e.g., Herrmannova and Knoth, 2016; Harzing and Alakangas, 2017; Hug and Brändle, 2017). However, MAG contains six entities that comprise Microsoft Research's model of scholarly activities: field of study, author, institution, paper, venue, and event (Sinha et al., 2015), from which other information about the publication is derived. Microsoft Research uses machine learning techniques to identify and extract “publication” as the primary entity in its academic entity-relationship graph, to which other entities are then related. Additional attributes of these six entities are then derived algorithmically. In order to disambiguate author names, Microsoft uses machine learning as well as crowdsourced data and assigns unique author IDs (Wang et al., 2019).

The MAG bibliometric data was collected via the CADRE PostgreSQL database and exported into a spreadsheet for data visualization, statistical analysis, and comparison. The query of the CADRE PostgreSQL database was designed so the columns representing authors and institutions, along with their corresponding publication IDs, could be parsed and converted into multiple rows. Each row of data contains an author, institution, and article identifiers. Thus, if an article has seven authors, there are seven rows of data in our MAG CSV file to represent it. Since each author was identified with a unique ID, no further author disambiguation was performed. Each unique author ID was tied to an author name and an institutional affiliation. Authors were associated with institutions on a per publication basis, so authors whose affiliations changed did not in any way compromise the accuracy of the connection between author and institution.

**FIGURE 1:**
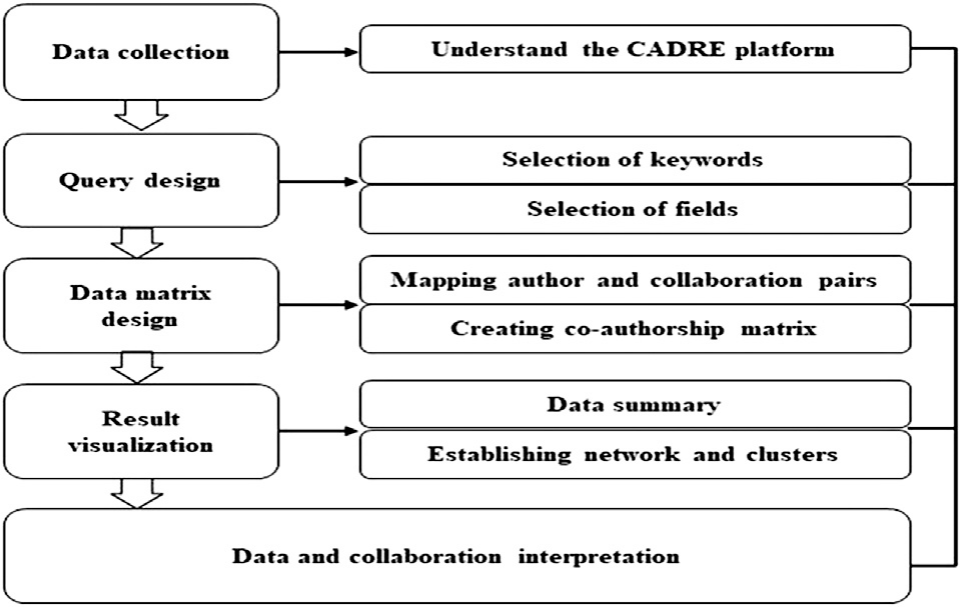
Steps of collaboration analysis used in SDG-related research.

There are limitations to the usefulness of bibliometric data from abstract and citation databases of peer-reviewed research literature brought on by incomplete coverage and the difficulties of author disambiguation, in this case, performed prior to our study by MAG algorithms. The methodology, including interpretation of the different metrics, builds on best practices on research evaluation developed throughout the years on quantitative science and technology studies (Moed et al., 1985; Moed et al., 2005). Figure 1 shows the steps taken by our study.

### Search Strategy and Validity

Because our study analyzes research collaborations that focus on Sustainable Development goals, our query includes both “sustainable development goal(s)” and “millennium development goal(s)” since these two phrases are conceptually and historically connected (Meschede, 2020). The Millennium Development Goals were derived from the United Nations Millennium Declaration, which was adopted by the Assembly in September 2000. However, some UN reports that addressed sustainable development goals were published in 1999. For this reason, our 20-year window of data extraction runs from 1999 to 2018 and captures all available publication types (article, review, conference papers, short survey, notes, book/book chapter, etc.). Our dataset includes the following fields: author name, author ID, document title, publication year, document type, affiliation name, and affiliation ID, extracted from the CADRE platform as CSV files. “Researchers” or “authors,” and “organization,” “affiliation,” or “institution” are used interchangeably in this study to describe the author and institutional profiles, respectively.

### Collaboration Metrics and Analysis

For this study, we focused our analysis of collaboration on authors and institutions, using co-authorship data as a proxy measurement. Publications with at least two unique authors (author-level analysis) and publications with at least two unique institutions (institution-level analysis) indicate collaboration for our purposes. Since we were identifying collaborations and repeat collaborations, not individual researcher impacts, we used “full counting,” which straightforwardly assigns full weight to each co-author on a publication, rather than fractional counting, in which each co-author is accorded weight as a fraction of the number of co-authors (Perianes-Rodriguez et al., 2016).

**FIGURE 2:**
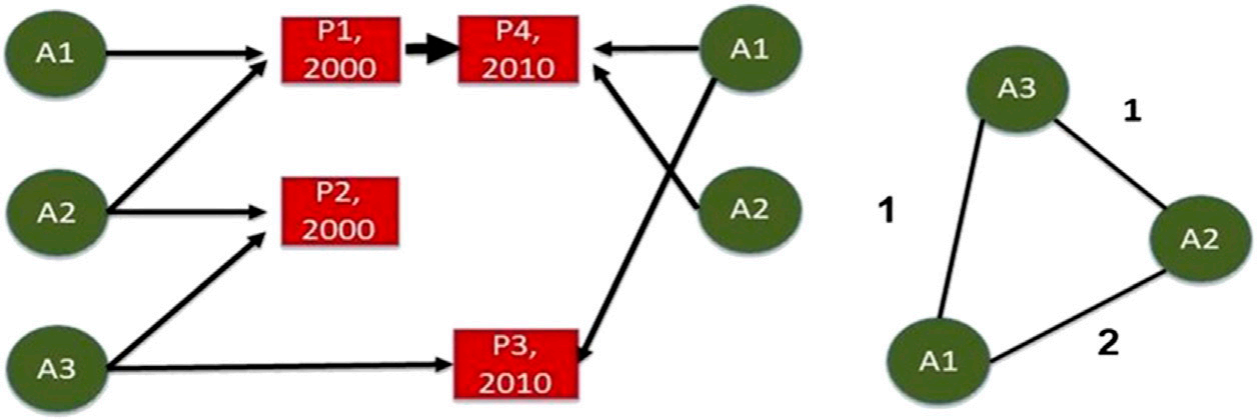
Illustration of co-authorship links shows three authors and four documents over time (left), and the construction of authorship networks using full counting (right).

To simply illustrate (Figure 2), we have three authors, labeled A1, A2, and A3, and four papers labeled P1, P2, P3, and P4. P1 is authored by A1 and A2, P2 is authored by A2 and A3, and P3 is authored by A1 and A3. Using the full counting two-mode network, the link between A1 and A2 has a strength of 2. This indicates that A1 and A2 have co-authored two papers, P1 and P4, in different years. This study was focused on determining this total link strength (how many times a given collaboration happened) and on the intervals between each collaborative output.

Table 1 presents the metrics used in this study. Descriptive statistics (e.g., frequency in count and percentage) of each of these metrics were collected. Changes across time were evaluated using linear regression while the Pearson correlation coefficient (Pearson’s *r*) was calculated to determine relationships between level of evidence and time, and the number of publications, unique authors, and unique institutions.

**TABLE 1:** Metrics used in profiling research outputs and collaboration in SDG-related research.

Metric	Description
Scholarly output	Number of SDG-related papers
Authors per paper	Average number of authors
Collaboration rate	Yearly percentage of publications with two or more authors (institutions) each year, categorized as domestic or international
Collaboration growth	Compound annual growth rate (CAGR) or year-on-year constant growth rate over a specified period
Repeat collaboration	Publications with persistent ties or repeated association among authors (or institutions)
Collaboration time point	Number of years for the repeat collaboration
Collaboration network	Illustrates scientific collaboration patterns between authors and institutions with repeat collaboration

### Network Analysis and Visualization

In addition to bibliometric analysis, we used several network analysis tools to visualize and measure connections between researchers and institutions engaged in SDG-related research. We used Jupyter Notebooks (Kluyver et al., 2016) to parse and analyze our data and generate network graphs to visualize relationships between researchers and between institutions in our dataset. The output was visualized with Flourish. Flourish was selected as a visualization tool because of its user-friendly interface to create interactive web-based graphs suitable for exploration. Since CADRE uses Jupyter Notebooks to bundle code and provide an interface for CADRE data, utilization of this tool helped us embed this work in a wider collaborative community. The CADRE team was able to use this code to create a reproducible package, making it easier for other members of the bibliometric community to try out the code and generate similar visualizations to the ones generated for this study.

Since the extracted MAG data was organized into comma-separated rows for each co-author (or institution) of a given publication, it was straightforward to read our article data into network graphs defining co-authorship. To create an authorship network graph, we used the python package NetworkX to build the graph structure (Hagburg et al., 2008). To visualize the exported output, we used the Flourish platform, for which we prepared two files, containing nodes and edges respectively, in tab- and comma-delimited files. To each of these data files, we added columns containing additional information derived from the initial dataset about each of the entity-nodes contained in the graph. These additions add context, help develop the graph's visual rhetoric, and provide pathways for viewers to explore features of the underlying data. The co-authorship graph, for instance, allows viewers to see a list of DOIs of SDG articles attributed to each author, as well as the author’s most recent institutional affiliation, as recorded in the MAG dataset. To the co-institutional graph, analogously, each institutional node is supplemented with listings of all affiliated authors and articles, as well as several pertinent statistical measures.

We also added a locational context in the form of the institution’s country and continent. This geographic information was gathered via the Google Maps Geocoding API and may contain inaccuracies but a manual review of a sample of the data suggests it is largely accurate. Having geographic information about each institution further allowed us to characterize each collaborative link between institutions as “domestic,” or “international” or “regional”, a basic distinction that helps reveal in which cases collaborations happen across national or continental boundaries, despite the difficulties those distances would seem to entail. By domestic collaboration, we refer to collaborations that involve multiple authors affiliated with institutions in the same country while international collaboration involves authors whose affiliated institutions are in different countries and regional collaboration involves authors whose affiliated institutions are between nearby countries. The collaboration pattern of institutions, how this research network is structured, and how the network clusters changed over time (1999–2008 vs. 2009–2018) were also visualized using Flourish. The full data from these graphs along with links to an interactive network graph is available via GitHub (https://github.com/iuni-cadre/Fellow3-MCAP).

To understand the overall characteristics and structure of the network, that is, the interconnectedness between the researchers and institutions represented in our data, network density, and transitivity were computed using NetworkX. To better understand the separable communities of collaboration at play in our network, modularity classes were computed using NetworkX’s implementation of the community detection algorithm developed by Clauset et al. (2004).

Degree, closeness, betweenness, and eigen centralities were also computed using the NetworkX package. These measures were deemed useful for describing the co-institutional network, but not the co-authorship network because of the latter’s very low density. Pathways between authors were too attenuated or broken to yield reliable centrality measures across the graph. The measurements were again computed on the version of the institutional graph which eliminates edges not represented by a weight greater than one.

Bibliometric networks were analyzed using centrality measures guided by processes described in Yan et al. (2010); Benckendorff (2010) and Abbasi et al. (2012). We used these measures because we are interested in the individual position and the importance of any given node within the network and over time. The Degree Centrality (DC) of a node is a count of its edges divided by the number of other nodes in the network. A high DC value for an organizational node would tend to indicate a higher centrality to the graph as a whole, but that determination would also depend on the overall shape of the network. Betweenness Centrality (BC), on the other hand, is the proportion of shortest paths between nodes in the network that pass through a given node. A high BC in our instance may indicate organizations that have more potential to influence the flow of research resources, and thus research priorities, in the network. The Closeness Centrality (CC) of a node is the reciprocal of the total distance from itself to all other nodes. A high CC value indicates that a node is very close to other nodes, suggesting that the organization with the highest closeness centrality has more ability to connect with other members of the network. Like DC, Eigen Centrality (EC) measures a node's influence based on the number of links it has to other nodes in the network. It measures how well connected an institution is, how many links the connections of this institution has, and so on through the network. A high EC for an organization means that the institution has many institutional connections and is connected to institutions with wide-reaching influence within a given network. Each of these measures was calculated using NetworkX, and the 20 institutions with the highest values were analyzed further.

To understand the change in repeat collaboration patterns of institutions over time, we divided the 20-year MAG dataset into two periods. Two additional institutional graphs were produced using the same methods as above, each representing a period of 10 years (1999–2008 and 2009–2018).

## Results

The initial search for the phrase “Sustainable Development Goals” resulted in more than 12,400 papers authored by more than 27,900 unique authors and more than 4,100 unique institutions. Expanding the search to include the phrase “Millennium Development Goals” added more than 5,200 papers, resulting in a dataset with 16,447 papers representing 35,333 unique authors and 4,656 unique institutions. We used the dataset from our expanded search query for this analysis. Table 2 provides more information on the basic features of the dataset analyzed for this study.

**TABLE 2:** Summary statistics by year.

	Total count (1999–2018)	Yearly mean	Std dev	Minimum	Maximum
Publications	16,447	822.35	615.07	71	1,971
Authors	35,333	2,437.75	2,561.78	127	8,953
Institutions	4,656	612.4	580.88	30	1,962

### Growth in Sustainable Development Goal Research

**FIGURE 3:**
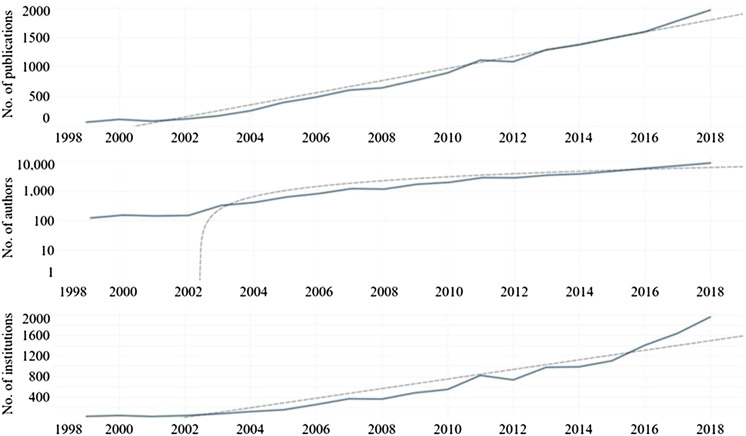
Growth in the rate of research output investigating SDGs over time (1999-2018) analyzed at the author and institutional levels.

Over the 20-year period covered by the data, publication output grew at a compound annual growth rate (CAGR) of 19%. A linear correlation with a coefficient of determination of 0.97 was found with the yearly number of research outputs and year. The increase in publications per year was statistically significant at the 0.0001 level. The highest publication count was in 2018, while 1999 had the lowest. The data also exhibit a similar growth rate for the number of authors (22.53%) and institutions (17.38%) over this period. Linear correlations with coefficients of determination of 0.90 and 0.97 were found between the yearly number of unique authors or institutions and year. Figure 3 shows the SDG-related research outputs from 1999 to 2018.

### Co-Authorship and Repeat Co-Authorship

Over the 20 years represented by this data, the average number of authors per paper has increased two-fold, from ∼2 authors per paper in 1999 to ∼4 authors per paper in 2018. The increase in unique authors and institutions collaborating per year was statistically significant at the 0.0001 level. The percentage of publications with multiple authors was 57.60% (9,470/16,447) while the percentage of publications with multiple institutions was 31.34% (5,155/16,447). Collaboration on SDG-related research has grown steadily at both the author and institutional levels. At the author level, an increase in collaboration rate was observed from 42.25% (30/71) collaboration in 1999 to 75.55% (1,489/1,971) collaboration in 2018. Similarly, an expansion in collaboration rate was observed for institutions from 12.68% (9/71) collaboration in 1999 to 44.29% (873/1,971) collaboration in 2018.

**FIGURE 4:**
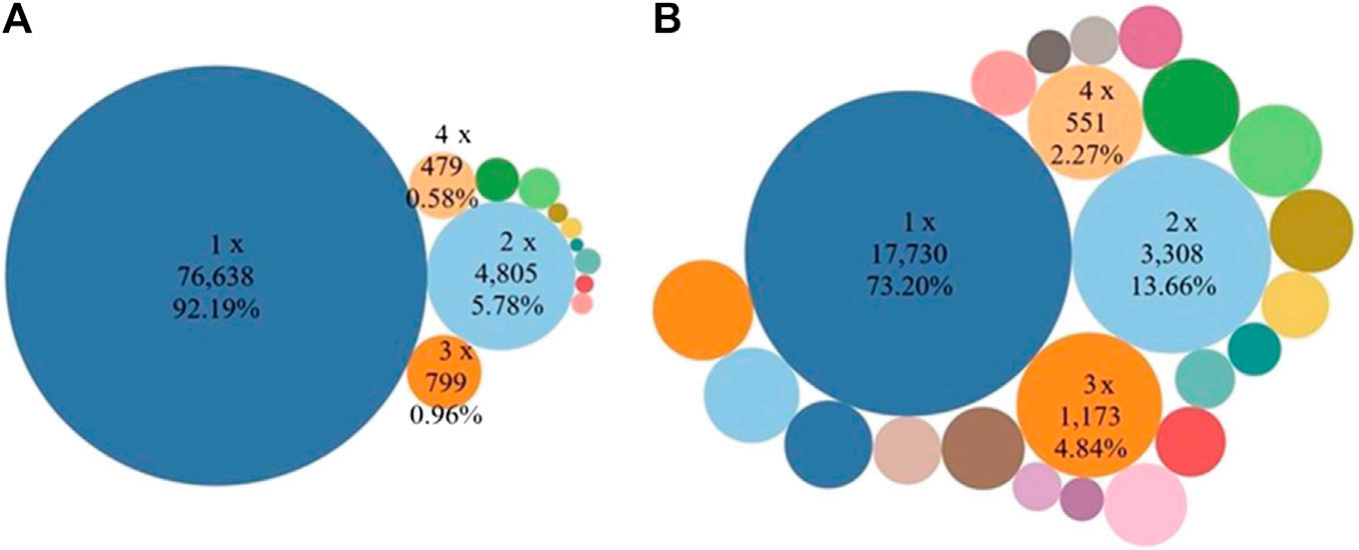
Co-authorship and repeat collaboration in SDG-related research among **(A)** authors and **(B)** institutions, 1999–2018. Most SDG-related research co-authorship happened only once.

We then narrowed the focus to only research outputs that were co-authored to look for indicators of repeat collaboration. Limited instances of repeated collaboration were observed, however. The bulk (92.19%) of authors collaborated only once, representing 73.20% of institutions with only one co-authored output. In addition, at the author level, 8.12% (769/9,474) of the collaborative papers involved repeated collaboration, while 27.27% (1,406/5,155) involved repeated collaboration at the institutional level. Our results showed that authors and institutions typically collaborated only once in most cases (Figure 4).

The percentage of authors collaborating only once was consistently high over the 20-year period and this percentage barely changed, from 88.89% (176/198) in 1999 to 87.33% (16,810/19,248) in 2018, decreasing at a 20-year CAGR of just 0.09%. An increase in collaboration was observed, on the other hand, when data was analyzed at the institutional level. The percentage of institutions collaborating only one time compared to the total number of co-authored publications has decreased from 45.59% (31/68) in 1998 to 18.42% (4,273/23,194) in 2018, indicating an increase in *institution* x *institution* collaboration over time. This is represented by a negative CAGR rate of 4.82% over the 20 years. The change in the proportion of institutions collaborating only once was statistically significant at the 0.0001 level.

### Temporal Structure of Sustainable Development Goal Research Collaboration

**FIGURE 5:**
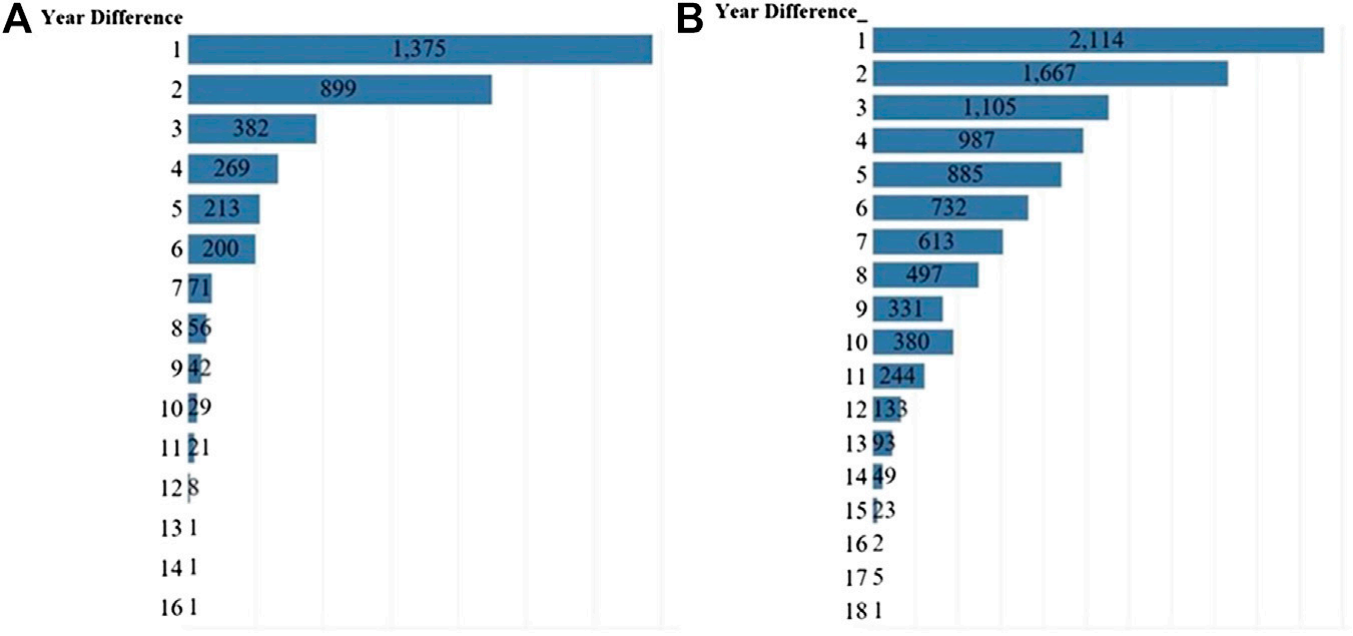
The temporal structure of collaboration among authors and institutions **(A)** years until the second co-authored publication **(B)** years until the second co-institutional co-authored publication. In our data, most repeat collaboration occurred after a year. Median repeat collaboration for authors was 2 years and for institutions was 4 years.

We also analyzed the temporal component of SDG research collaboration (Figure 5). Researchers who collaborated again with a prior co-author did so after two years (mean = 2.7 years; median = 2 years) and institutions collaborated again with the same institution after four years (mean = 4.38 years; median = 4 years). More than 38% (1,375/3,569) of unique *author* x *author* combinations and 21.60% (2,163/10,012) unique *institution* x *institution* combinations collaborated again within a year.

### Sustainable Development Goal Collaboration Networks

To further characterize SDG research collaborations, we then turned to network analysis to characterize SDG research networks.

**FIGURE 6:**
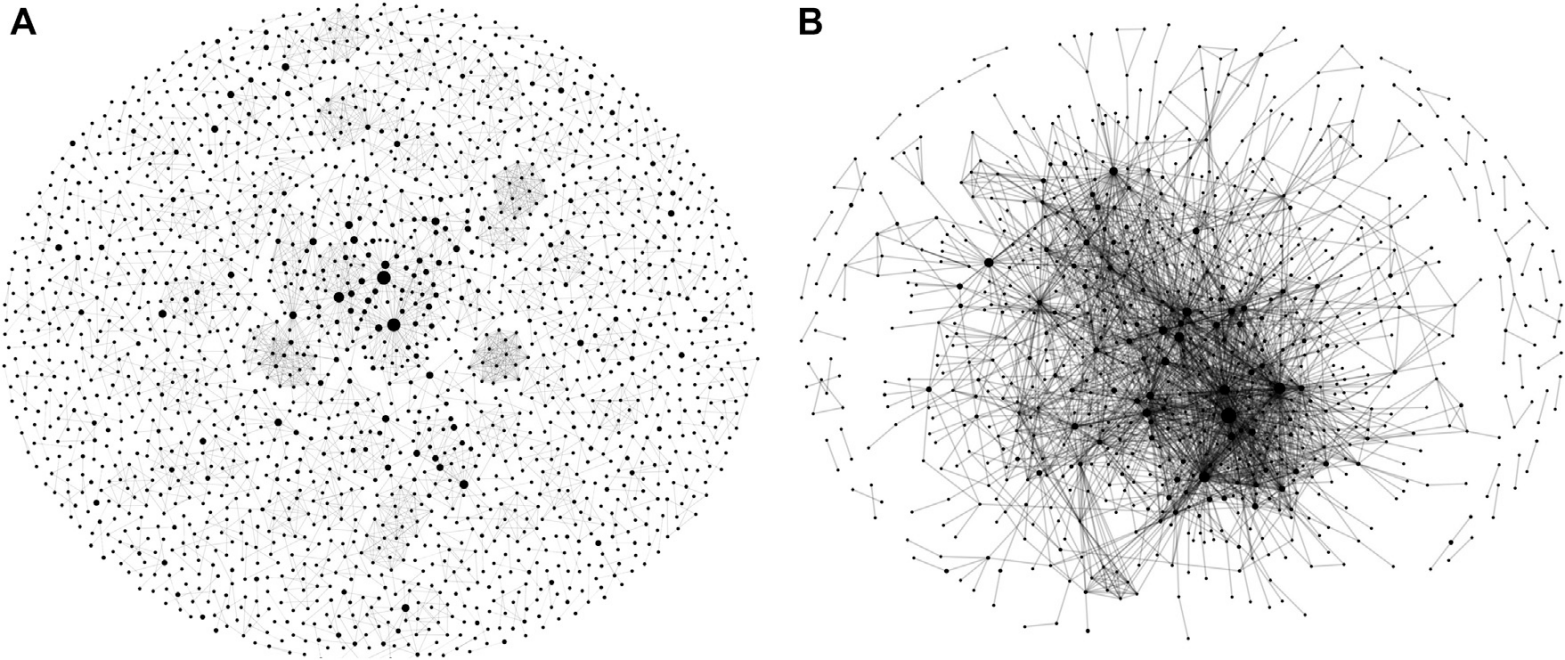
Wide-scale representations of **(A)** author, and **(B)** institutional graphs. Each circle, or node, in these and other network graphs visualized in this study, represents an author/institution, and lines between circles represent co-authorship. The size of the circle corresponds to the number of articles published by that author/institution within our dataset. The thickness of the line represents the frequency of the co-authoring. Interactive versions of these and the following graphs are available via GitHub (https://github.com/iuni-cadre/Fellow3-MCAP).

**FIGURE 7:**
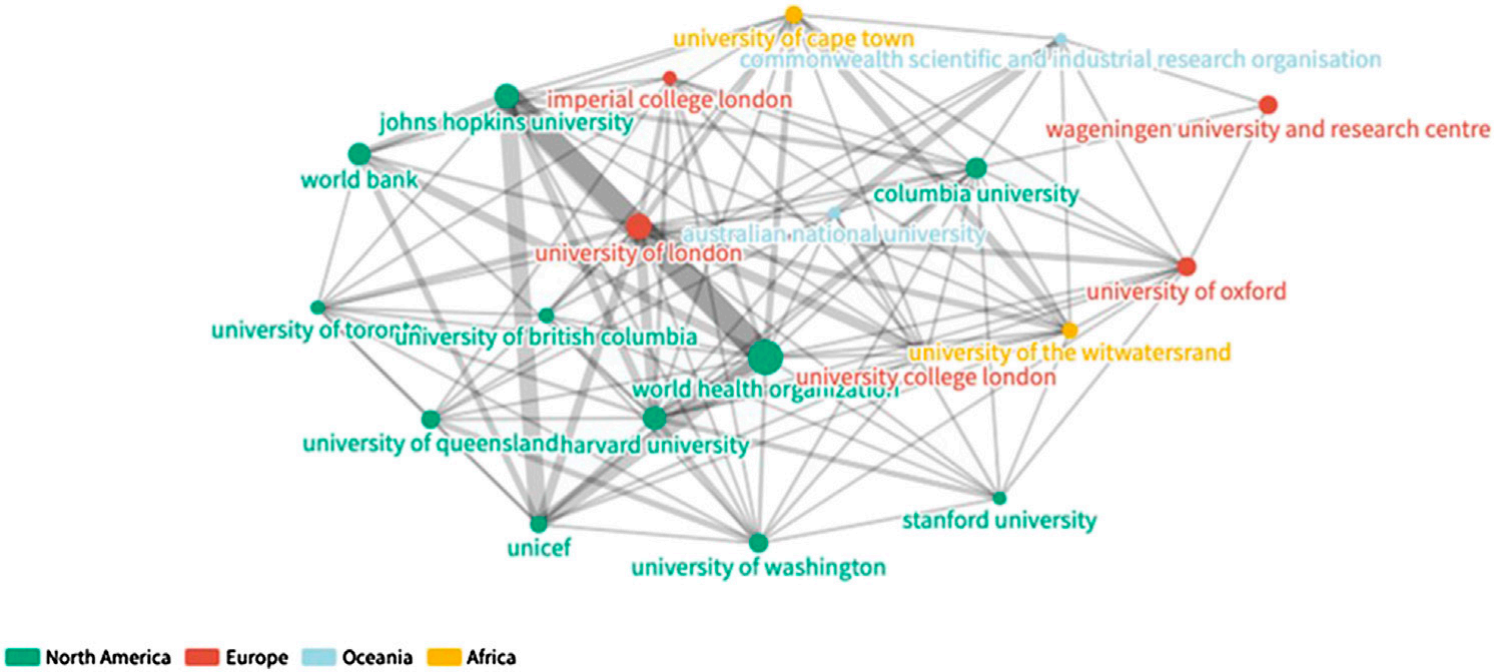
SDG-research collaboration showing institutions with highest degrees, 1999-2018.

Table 3 provides an overview of these networks, and the graphs in Figure 6 provide a bird’s-eye view of the complete collaborative networks. These overviews highlight the relative shape and density of the author and institutional graphs, respectively, which show distinctive structures. The author graph of Figure 6A remains quite diffuse, with small groups of collaborating authors at times branching out to form larger clusters with other groups. Repeated partnerships are also visible in constellations of 6–20 authors forming complete mini-graphs unto themselves. The tightness of these localized clusters is reflected in the author graph's higher transitivity (0.6756) than the institutional representation (0.1876). The institutional graph of Figure 6B, by contrast, makes visible how those same collaborations also reflect a highly centralized structure, congested with dense institutional interrelationships in a highly developed center, which then ramifies out into series of less well-connected institutions primarily joined to the network via their link to the brokers of the central hub. Figure 7 depicts just those nodes in the institutional graph with over 40 connections, thus, with a degree over 40.

**TABLE 3:** Descriptive statistics for Institutional Graphs.

Institutional graphs by time period (edge weight >1)				Author graph (edge weight > 1)
Period	1999–2008	2009–2018	All years	All years
Number of articles	3,012	13,435	16,447	16,447
Node count	101	751	828	1,913
Edge count	145	2,089	2,389	2,847
Average degree	2.762	5.563	5.769	2.977
Density	0.0266	0.0074	0.0071	0.0016
Transitivity	0.3671	0.1926	0.1876	0.6757
Components	20	49	47	517
Nodes in largest component	56	643	572	288
% Domestic	39%	42%	41%	Not calculated
Communities (largest component)	5	16	16	14

Both graphs in Figure 6 display only repeated collaborations, that is, those edges with a weight greater than one. The sizes of the nodes in both graphs are scaled according to the number of publications associated with that author or institution from across our dataset, not necessarily the extent to which either may have contributed to co-authorship; that is, single-authored publications are figured into the size of the node. The full data from these graphs along with links to an interactive network graph is available via GitHub (https://github.com/iuni-cadre/Fellow3-MCAP).

### Network Centrality and Clustering

Given the relative disconnectedness of the author network, much of the subsequent analysis focuses on institutions that co-originated two or more publications. Table 4 presents the aggregated centrality measures and descriptive statistics for these institutions while Table 5 presents the institutions that consistently ranked highest in all centrality measures. The average scores of the institutions for all centrality measures ranged from 0.0000 to 0.3865 while SD ranged from 0.0082 to 0.0921.

**TABLE 4:** Descriptive statistics for network measures.

	No. of institutions	Mean	Std dev	Minimum	Maximum
BC	828	0.0024	0.0082	0.0000	0.1183
CC	828	0.2152	0.0921	0.0012	0.3865
DC	828	0.0070	0.0133	0.0012	0.1439
EC	828	0.0155	0.0311	0.0000	0.2847

**TABLE 5:** Centrality measures for institutions that consistently ranked highest and with two or more co-authored publications.

Institution	BC	CC	DC	EC
World health Organization	0.1183	0.3865	0.1439	0.2691
University of London	0.0893	0.3850	0.1415	0.2847
Harvard University	0.0658	0.3728	0.1076	0.2424
Johns Hopkins University	0.0643	0.3648	0.1209	0.2394
Columbia University	0.0608	0.3735	0.0859	0.1928
Commonwealth Scientific and Industrial Research Organization	0.0493	0.3382	0.0641	0.0889
University of Washington	0.0437	0.3544	0.0750	0.1789
Wageningen University and Research Center	0.0432	0.3104	0.0641	0.0546
Australian National University	0.0421	0.3425	0.0556	0.1040
University of Cape Town	0.0337	0.3494	0.0508	0.1393
Imperial College London	0.0320	0.3516	0.0580	0.1399
University College London	0.0308	0.3479	0.0580	0.1498
University of Melbourne	0.0302	0.3457	0.0459	0.1333
Stanford University	0.0283	0.3502	0.0617	0.1365
University of British Columbia	0.0268	0.3316	0.0508	0.1007
University of oxford	0.0250	0.3469	0.0556	0.1288
World Bank	0.0248	0.3342	0.0520	0.1200

The World Health Organization held the highest BC, the highest CC, and the highest DC. The University of London, on the other hand, held the highest EC. The top three institutions in terms of BC and EC include the World Health Organization, the University of London, and Harvard University. In terms of highest BC, the World Health Organization, University of London, and Columbia University while in terms of highest DC, World Health Organization, University of London, and John Hopkins University landed in the top three.

**FIGURE 8:**
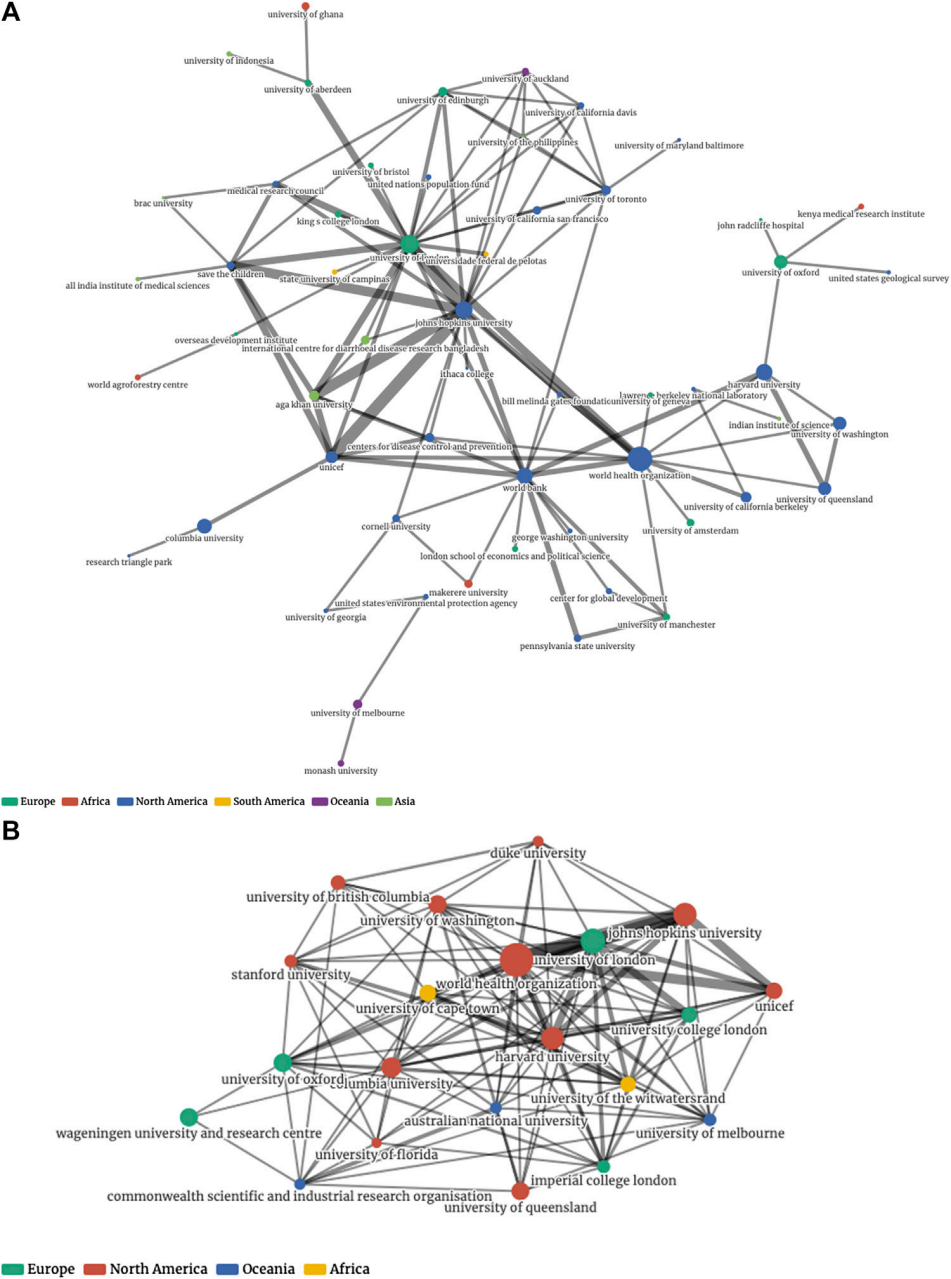
Representations of institutional networks by time period: **(A)** Repeat collaboration network, 1999–2008. **(B)** Top 20 nodes by degree, 2009–2018.

**FIGURE 9:**
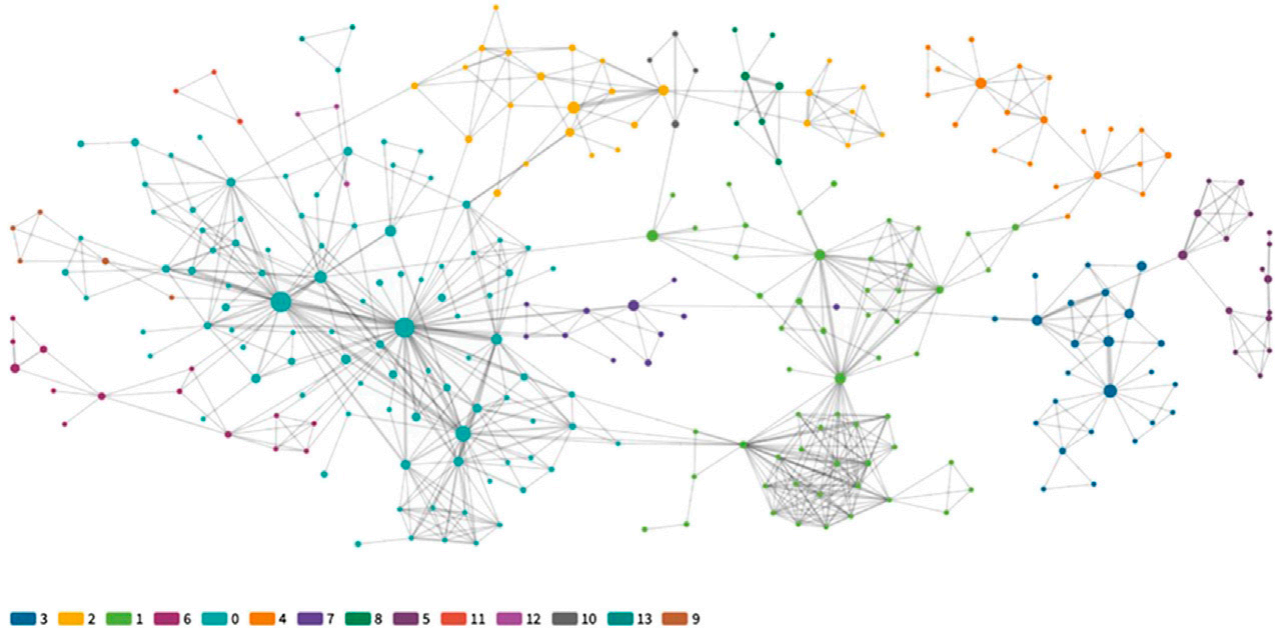
The single largest component of the author network, with communities indicated by color.

**FIGURE 10:**
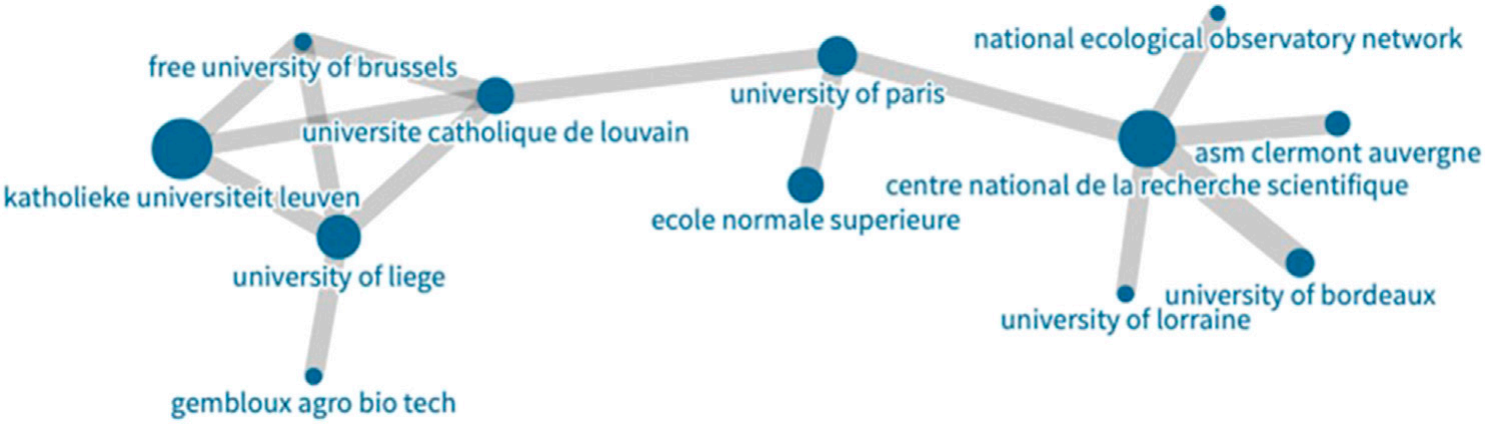
Community two from the largest component of the complete graph of institutional collaboration (Figure 6B). This community is not connected strongly to the central hub of large influential institutions and shows that geographic proximity also defines collaborative communities in SDG research.

Figures 8A and B provide a visual comparison (1999–2008 vs. 2009–2018) of the research networks established in SDG-related research over the years. These visualizations show the prominence of several large organizations. As shown in Table 3, 572 out of 828 organizations belong to one large central community. Figure 9, by contrast, provides a visual illustration of the largest component of the network of authors with repeat coauthorship. Here a set of smaller networks interconnect to form a larger one not dominated by any central hub, reflecting smaller but overlapping author groups. Looking again at the institutional network, all of the minor communities are to some extent defined by regional or linguistic ties. Figure 10 shows one such smaller linguistic and regional community of Belgian-French co-authorship that has not yet been strongly tied to the major organizations at the center of the larger institutional graph.

## Discussion

This study deployed bibliometric and network analysis to explore the emergence and patterns of collaborative research investigating the United Nations Sustainable Development Goals—a concerted effort to meet global challenges without depleting the environment beyond its capacity to regenerate. Research collaboration, networks, and partnerships are essential mechanisms for meeting SDG targets (Stibbe et al., 2018; HESD-IAU, 2020) and sustainable development (Sonnenwald, 2007). Specifically, this study uses the author and institutional affiliations extracted from Microsoft Academic Graph to analyze the collaboration network in this topical area at the author and institutional levels. In addition to traditional collaboration metrics (e.g., co-authorship links), we supplemented this analysis by mapping other characteristics of research collaborations (e.g., growth, frequency, length, and network) to examine collaborations and partnerships supporting SDG research.

### In Our Analysis

(1) Collaboration on SDG-related research reveals trends of accelerating collaboration rate over time at the author and institutional levels.
(2) The frequency of collaboration on SDG-related research reveals that the majority of collaborations in SDG-related research happened only once.
(3) Co-authorship data show that repeat collaboration for authors typically occurred at 2 years; for institutions, this happened after 4 years.
(4) The network of repeated institutional collaboration reveals a highly centralized structure. About a third of institutions with repeated collaborations are U.S.-based, a far higher proportion than any other country.
(5) Large organizations with global reach dominate the structure of the network, but clusters of regional collaboration, that is, collaborations between groups located in nearby countries, are also in evidence.
(6) Our analysis of the SDG research network identifies candidate institutions that are well-placed to help generate more collaboration and sustain the research network.

Through this study, we confirmed that SDG research is growing, as measured by the number of documents published annually, demonstrating increasing interest in sustainable development goals within the research community. This result also confirmed the growing knowledge base on SDG-related research which we cited in this work.

Collaboration was also on the rise, with more authors and institutions collaborating over time, indicating that collaboration is becoming a mainstream approach for this area of research. The overall increase in collaboration for SDG-related research is reflected in other analyses, including Sustainability Science (2015) and Nakamura et al. (2019), which both examined the state of sustainability science and the collaboration supporting it. This upward trend of collaboration at both author and institutional levels may be associated with the combination of changes cited by other studies from the social organization of the scientific community to better communications (Jeong et al., 2014), increased research funding, and access to other resources, e.g., facilities and labor (Chang and Huang, 2016), and possible changes in the type of research questions studied and approaches used (Adams, 2012; Wagner, 2018), making globally relevant topics like SDG more amenable to collaborative investigation. Specific factors for fostering collaboration on sustainable development goal-directed research bear further investigation.

While the collaboration rate is increasing, nevertheless, results point to the presence of multiple one-time collaborations and less common instances of repeat collaborations among both authors and institutions during the 1999–2018 period. On the one hand, this limited co-authorship does not take advantage of the anticipated effects of repeat collaboration on coordination, communication, and task routines that improve performance (Bercovitz and Feldman, 2011), but suggests other motivations or constraints for researchers. Skilton and Dooley (2010), for instance, indicated that pre-existing relational ties result in diminishing research creativity and performance among researchers. Our results also showed that when authors had repeat collaborations, those collaborations happened sooner rather than later. This follows on from the common-sense intuition that one would tend to continue co-authoring with those with whom they are personally acquainted (Newman, 2001) and one’s more recent collaborators than those in the more distant past.

The rate of repeat collaboration was found to be greater at the institutional level than at the author level. Since institutions are aggregations of researchers, this implies more possibilities for institutions to connect via research partnerships. This strategy may be linked with institutional guidelines and policies for managing research (i.e., fund allocation, research impacts documentation, accountability, etc.) and in establishing alliances. Gazni and Thelwall (2016) show that not only has institutional collaboration increased over time, but it has increased more quickly for high-impact institutions, pointing to the ability of researchers at these institutions to be influential in their choice of collaborating partners. Many groups, including the International Association of Universities (HESD-IAU, 2020) advocate for institutional collaboration in meeting sustainable development targets. Over time, an increase in institutional participation in repeat collaboration and an increase of collaboration within the scientific network for SDG-related research were also noted. It bears investigation into how initial partnerships to study SDGs relate to later partnerships at the institutional level, for example, how universities renew international agreements and memoranda of understanding, assess or reward cross-institutional research productivity, or otherwise assess whether the benefits outweigh the costs.

The scientific collaboration network structure of repeat collaboration was central over time at both author and institutional levels. This central structure indicates that scientific knowledge is concentrated in the focal points of the network. About a third of institutions with repeated collaborations are United States-based, a far higher proportion than any other country. The United Kingdom, in second place, is home to about 8% of institutions with repeat collaborations. Europe, taken as a whole, accounts for 30% of institutions included in our data. Nakamura et al. (2019) tallied regional patterns of collaboration in SDG research and reported that European institutions had higher levels of bilateral and multilateral international collaboration for SDG research, with the United States and other regions contributing less. United States-affiliated authors on SDGs do not yet engage in large, stable networks. These results contrast Sweileh (2020) who found increases in co-authored publications across all SDGs for the United States. United States institutions tend to collaborate more frequently and have more opportunities to co-author papers in SDG-related research compared to other institutions, and hence have more opportunities to create a larger scientific network supporting SDG research. Our results complement Sweileh’s result and suggest that the United States is more active in repeat collaboration than European nations and other countries in SDG-related research.

Pursuing and maintaining successful research collaborations take time and our author and institutional analyses point to the persistence of United States-affiliated authors and institutions to pursue and endure longer-term collaboration with tangible outputs and potential impact in SDG-related research. The United States has the largest number of global diaspora members of any country in the world (Payumo et al., 2017), which enables international development and knowledge transfer; this too may have been a factor in repeat or continuous collaborative efforts between the U.S. and other institutions across the globe.

Approaches that address health are key to achieving the SDGs. Hence, it is not surprising that the World Health Organization, a specialized agency of the United Nations, topped the list of institutions with repeat collaboration and with high scores in all centrality measures: DC, BC, and CC. WHO coordinates the “Global Action Plan for Healthy Lives and Well-Being for All” (World Health Organization, 2020), and leads the global effort on disease monitoring, as demonstrated by the ongoing SARS-CoV-2 pandemic. With the exception of WHO and The World Bank, all the top institutions highly engaged in repeat collaboration represent universities. Universities can serve as engines of transformation, and SDGs, along with their targets and indicators, can be used as measures to assess the impact and demonstrate the commitment to meeting societal challenges (Aurora Universities Network, 2020). Given the role of universities as partners in meeting global challenges, the inclusion of SDGs as a metric in university rankings is justified (THE Impact Ratings, 2020).

The network of SDG institutional collaborations indicates the importance of the brokerage of several large organizations. Since these organizations are primarily located in the United States and Europe, but with global reach, international collaborations are frequent. Segmenting the graph into communities reaffirms that collaborations anchored by these global organizations define one mega-community. Since the nature of institutional collaboration is that many separate groups may be operating independently, it is reasonable to hypothesize that even among those universities, companies, and other entities that are linked to the major organizations, domestic and regional collaborations are still in general preferred. Communities not defined centrally by connections to hub institutions exhibited more strongly regional ties. The influence of geographic proximity is still an important driver in the collaborative communities in SDG research, which corroborates the findings of Glänzel and Schubert (2005) and Benckendorff (2010).

The work of Payumo et al. (2019) suggests the major predictors of international research collaboration involving research-intensive universities. These predictors include: when collaborative research areas are linked STEM-related fields; when collaborative research involves a multidisciplinary field; when collaborative research is funded by an international grant; when opportunities for networking and peer-to-peer connections exist; when researchers involved in collaborative research have substantial international education and experience; and when there are publications and scholarly outputs involved for these collaborations. We anticipate that similar factors, especially co-authored publications and the scholarly impact of these publications apply when researchers and institutions pursue collaboration whether domestic or international and repeat these collaborations. It will be interesting to map this connection and conduct a case study focused on institutions engaged in repeat collaboration in SDG-related research that were identified in this study and determine factors that influenced repeat collaboration at the institutional and individual levels.

We note that the focus of this study was collaboration at the author and institutional levels, to characterize partnerships and identify factors in SDG-focused collaborations. This suggests a further study, through citation analysis, to understand the quantitative effect of repeat collaboration on scientific productivity and research impact in SDG-related research. Although the MAG dataset offered the advantage of coverage, it does not include citation data that would have permitted other kinds of analysis. CADRE anticipates future work incorporating and curating citation data for MAG-indexed publications, which we hope to utilize in a future study. This will enable an investigation to determine how citation data illuminates repeat collaboration in SDG-related research, and potentially test bibliometric perspectives and methodologies by other studies (e.g., Bu et al., 2017; Bu et al., 2018).

Finally, the pandemic due to SARS-CoV-2 and other unresolved crises linked to SDGs, and the inclusion of SDGs as one of the metrics in university rankings (THE Impact Ratings, 2020) raises more expectations for research collaborations; expanding this study in the future to determine the effect of the pandemic on research productivity, and other developments in the structure of collaborative science in SDG-related research would be a productive follow-up study.

While we recognize that this study has its limitations and caveats, we identified knowledge gaps in SDG-related collaborations and identified their characteristics and important features. The results from our analysis have implications for improving our understanding of the state of collaboration and measures for mapping stable, long-term partnerships.

## Data Availability

Publicly available datasets were analyzed in this study. The datasets used in this study can be found here: https://github.com/iuni-cadre/Fellow3- MCAP.
